# Comparative analysis of brain language templates with primary language areas detected from presurgical fMRI of brain tumor patients

**DOI:** 10.1002/brb3.3497

**Published:** 2024-06-19

**Authors:** Jina Lee, Vinodh A. Kumar, Jian Ming Teo, Rami W. Eldaya, Ping Hou, Kyle R. Noll, Sherise D. Ferguson, Sujit S. Prabhu, Ho‐Ling Liu

**Affiliations:** ^1^ Department of Neuroradiology The University of Texas MD Anderson Cancer Center Houston Texas USA; ^2^ Department of Imaging Physics The University of Texas MD Anderson Cancer Center Houston Texas USA; ^3^ The University of Texas MD Anderson Cancer Center UTHealth Graduate School of Biomedical Sciences Houston Texas USA; ^4^ Department of Neuro‐Oncology The University of Texas MD Anderson Cancer Center Houston Texas USA; ^5^ Department of Neurosurgery The University of Texas MD Anderson Cancer Center Houston Texas USA

**Keywords:** brain tumor, functional MRI, language, language template

## Abstract

**Introduction:**

Functional brain templates are often used in the analysis of clinical functional MRI (fMRI) studies. However, these templates are mostly built based on anatomy or fMRI of healthy subjects, which have not been fully vetted in clinical cohorts. Our aim was to evaluate language templates by comparing with primary language areas (PLAs) detected from presurgical fMRI of brain tumor patients.

**Methods:**

Four language templates (A–D) based on anatomy, task‐based fMRI, resting‐state fMRI, and meta‐analysis, respectively, were compared with PLAs detected by fMRI with word generation and sentence completion paradigms. For each template, the fraction of PLA activations enclosed by the template (positive inclusion fraction, [PIF]), the fraction of activations within the template but that did not belong to PLAs (false inclusion fraction, [FIF]), and their Dice similarity coefficient (DSC) with PLA activations were calculated.

**Results:**

For anterior PLAs, Template A had the greatest PIF (median, 0.95), whereas Template D had both the lowest FIF (median, 0.074), and the highest DSC (median, 0.30), which were all significant compared to other templates. For posterior PLAs, Templates B and D had similar PIF (median, 0.91 and 0.90, respectively) and DSC (both medians, 0.059), which were all significantly higher than that of Template C. Templates B and C had significantly lower FIF (median, 0.061 and 0.054, respectively) compared to Template D.

**Conclusion:**

This study demonstrated significant differences between language templates in their inclusiveness of and spatial agreement with the PLAs detected in the presurgical fMRI of the patient cohort. These findings may help guide the selection of language templates tailored to their applications in clinical fMRI studies.

## Graphical abstract

Composite of the templates overlaid on the MNI standard brain images in the anterior and posterior primary language areas

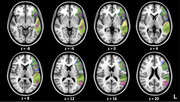

## 1 INTRODUCTION

Task‐based (tb) and resting‐state (rs) functional MRI (fMRI) are widely used to detect functional areas or networks in the brain by measuring changes in blood oxygen level–dependent signal in response to neuronal activities (Biswal et al., 1995; Ogawa et al., 1990). Clinically, fMRI is a routine procedure to localize eloquent cortices for presurgical evaluation of brain tumor resections (Lee et al., 2013; Matthews et al., 2006). Mapping and characterizing language areas is one of the most critical tasks for clinical fMRI, as the language function is an essential part of patients’ quality of life (Berger et al., 2020; Unadkat et al., 2019). However, this remains a challenging task as locations of language areas have been shown to vary significantly among patients (Sanai et al., 2008).

Functional brain templates are putative regions of interest (ROIs) in the brain that are responsible for specific functions (Laird et al., 2005). Language brain templates typically include anterior and posterior primary language areas (PLAs), and some include other language‐associated areas that are representative of a population of subjects (Fedorenko et al., 2010). After spatially transformed to an individual subject's space, such templates can help clinical fMRI analysis to evaluate language laterality from tb‐ or rs‐fMRI (Agarwal et al., 2018; Gohel et al., 2019; Pillai & Zaca, 2011; Ruff et al., 2008) and to guide seed selection for mapping the language network with rs‐fMRI (Hsu et al., 2020). In addition, previous studies have demonstrated the feasibility of categorizing functional networks from independent component analysis (ICA) of rs‐fMRI by using an automated template‐matching process and showed success in identifying language networks for presurgical mapping (Branco et al., 2016; Tie et al., 2014).

Multiple language templates from previous studies have been made publicly available for researchers (Caviness et al., 1996; Fedorenko et al., 2010; Lipkin et al., 2022; Shirer et al., 2012). They are mostly built based on brain anatomy, tb‐fMRI activations, or rs‐fMRI networks of healthy subjects. However, due to large intersubject variability in language regions (Frost & Goebel, 2012; Juch et al., 2005; Xiong et al., 2000), whether language templates generated based on a specific population can be applied to individuals of other populations should be inspected carefully. In patients with brain tumors, PLAs are particularly widespread and can extend beyond the classical boundaries of the Sylvian fissure (Sanai et al., 2008). Mass effect and reorganization of tumors (Dallabona et al., 2017) can additionally shift the PLAs from canonical functional anatomy. These causes of anatomic variability in the language function highlight the difficulty of finding a template for presurgical fMRI applications. To address this difficulty, this study aimed to evaluate and compare four publicly available language templates that have been used in previously published clinical fMRI studies (Branco et al., 2016; Gohel et al., 2019; Hsu et al., 2020; Zacà et al., 2018). Each template was analyzed for its ability to capture the maximum amount of PLA activation from presurgical fMRI of brain tumor patients, while minimizing the inclusion of activations that are not in the PLAs. In addition, spatial similarities between the templates and the PLA activations were evaluated.

## 2 MATERIALS AND METHODS

This retrospective study was approved by the institutional review board at our institution, and informed patient consent was waived. Initially, 40 patients with pathologically confirmed brain tumors who underwent language tb‐fMRI from November 1, 2021 to April 30, 2022 as part of their preoperative evaluation were identified and sequentially included in the study. One patient was excluded as this patient did not have significant (*p *< .05, corrected for family‐wise error [FWE]) PLA activation from any fMRI paradigm. A final total of 39 patients (22 males and 17 females; mean age, 48 ± 15.0 years) were included in the study. The mother tongue for all the patients was English. Patient demographics, tumor location, and tumor pathology are listed in Table 1.

**TABLE 1:** Patient demographic and clinical characteristics.

Characteristic	No. of patients
Age (mean) (range) (year)	48 ± 15.0 (14–78)
Sex	
Male	22
Female	17
Hand dominance	
Right	32
Mixed	5
Left	2
Tumor location	
Left frontal	16
Left parietal	5
Left frontoparietal	2
Left temporal	11
Left insula	1
Right frontal	2
Right temporal	1
Right insula	1
Pathology	
Glioblastoma	22
Oligodendroglioma	5
Grade II astrocytoma	4
Anaplastic astrocytoma	1
Diffuse astrocytoma	1
Ganglioglioma	1
Meningioma	1
Non‐Hodgkin lymphoma	1
Metastasis	2
Glioependymal cyst	1

### 2.1 Image acquisition

All patients received fMRI scans with multiple language task paradigms as part of their standard of care. Two of the paradigms, letter fluency (LETT) and sentence completion (SENT), were chosen for this study based on the recommendation of the American Society of Functional Neuroradiology Language Paradigm Taskforce (Black et al., 2017). Both paradigms included six cycles of 20‐s control and 20‐s task blocks. For LETT, patients were provided a letter of the alphabet and asked to covertly generate as many words as they could that began with that letter during each task block. For SENT, patients were asked to think of a word to fill in the blank that would complete the sentences presented during the task blocks. The paradigms were displayed with an MRI‐compatible, 32‐in.‐wide liquid crystal display (Invivo SensaVue, Phillips).

All MRIs were performed on 3T clinical scanners (GE Healthcare) that include the fMRI scans using a T2*‐weighted gradient‐echo echo‐planar imaging sequence (TR/TE = 2000 ms/25 ms; 32 slices with 4‐mm thickness and no gap; in‐plane resolution = 3.75 × 3.75 mm^2^). The anatomic images were acquired using a 3D T1‐weighted inversion recovery‐prepared spoiled gradient‐echo sequence and a T2‐weighted FLAIR sequence.

### 2.2 fMRI analysis

The fMRI data were processed using SPM12 software (https://www.fil.ion.ucl.ac.uk/spm/software/spm12/). Image preprocessing included motion correction, normalization to the Montreal Neurological Institute (MNI) space, spatial smoothing with a 6‐mm full width at half maximum Gaussian kernel, and high‐pass temporal filtering at a frequency of 1/128 s^−1^. Any fMRI scans with movement of >2 mm translation or >2° rotation in any direction were excluded. The spatial normalization was performed by co‐registering each patient's functional and T1‐weighted images and applying the deformation field obtained when normalizing the T1‐weighted images to the MNI space (see an example in Fig. s1). This approach along with the default normalization algorithm of SPM12 was found to perform reasonably well for registering brain atlas to patients with brain tumors (Chen et al., 2021). A functional activation map was then obtained by using the general linear model with the task paradigms convolved with a canonical hemodynamic response function. Each activation map was thresholded at *t* > 5.1 (*p *< .05, FWE corrected) as well as using the activation mapping as a percentage of local excitation (AMPLE) threshold (Gross & Binder, 2014) method. Only fMRI data with significantly activated clusters in the left hemisphere of the brain were selected for analysis. For the AMPLE thresholding, thresholds of 50% peak values were set separately for the anterior and posterior PLAs.

Two board‐certified neuroradiologists with expertise in clinical fMRI independently inspected the significantly activated areas obtained from the fMRI and manually outlined the ones in the anterior and posterior PLAs. The anatomical reference for anterior PLAs was the posterior inferior frontal gyrus, including the pars *triangularis* and pars opercularis. The anatomical references for posterior PLAs were the posterior superior temporal gyrus and posterior middle temporal gyrus. Variations in anatomy and fMRI cluster distribution were also considered on an individual patient basis. For any discrepancies, the two neuroradiologists reviewed them together to determine the areas by consensus. Masks of activations in anterior and posterior PLAs were then generated for each fMRI scan using the Multi‐image Analysis GUI software (https://mangoviewer.com/index.html).

### 2.3 Comparison of language templates and fMRI in PLAs

**FIGURE 1:**
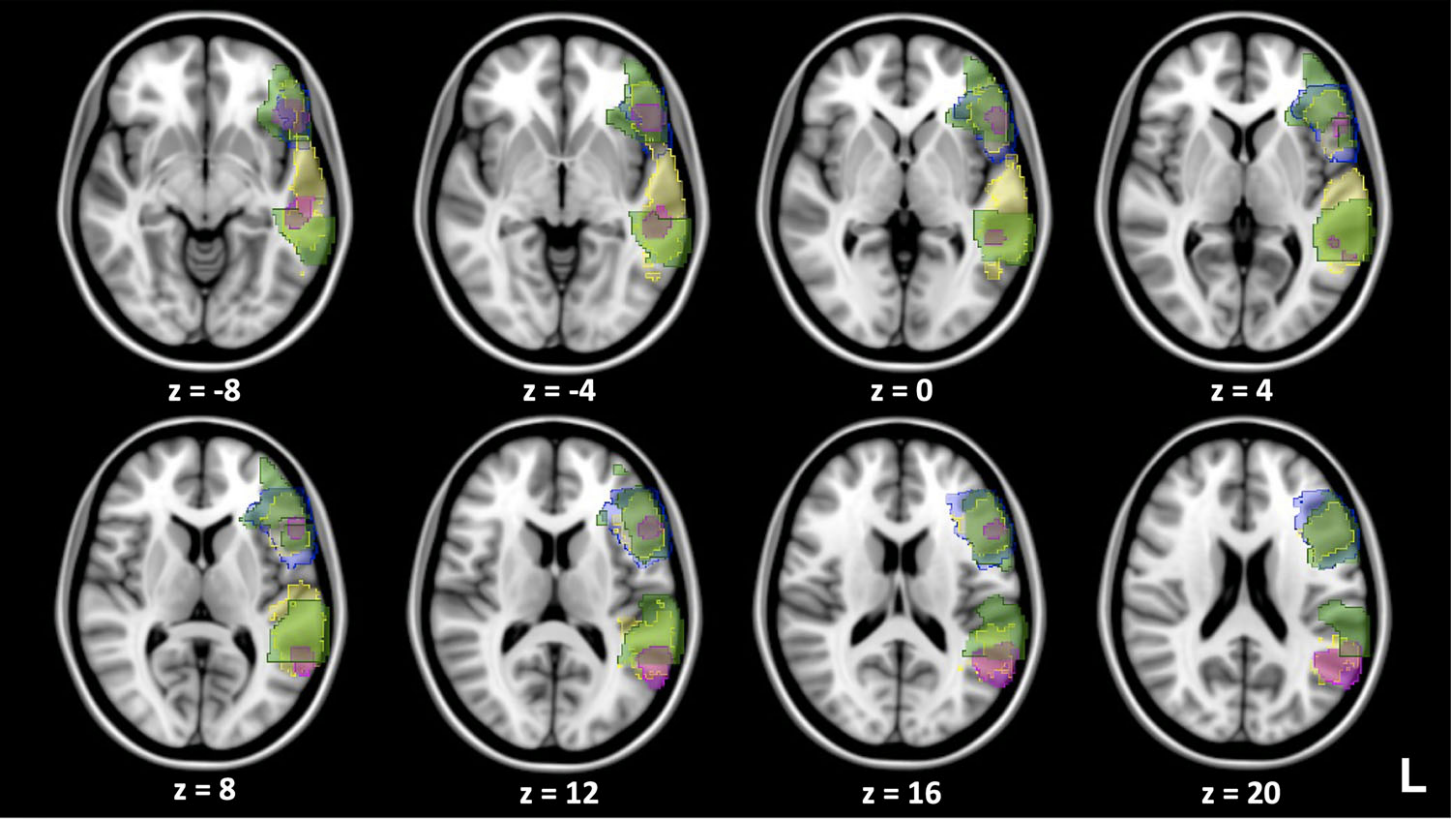
Composite of the templates overlaid on selected axial slices of the Montreal Neurological Institute (MNI) standard brain image in the anterior and posterior language areas. Blue: Template A, green: Template B, pink: Template C, yellow: Template D.

Four language templates that had previously shown success for applications in presurgical language mapping were selected for this study: (1) anatomically determined with Harvard–Oxford Atlas (Caviness et al., 1996) (Template A), (2) generated based on tb‐fMRI study (Branco et al., 2016; Fedorenko et al., 2010) (Template B), (3) generated based on rs‐fMRI study (Shirer et al., 2012; Zacà et al., 2018) (Template C), and (4) obtained from meta‐analysis results from Neurosynth with anatomical constraints (Hsu et al., 2020; Yarkoni et al., 2011) (Template D) (Figure 1). More details about the construction of these atlas can be found in Section 4. For each template, only ROIs in left hemisphere were included for the comparison. In addition, for the templates having multiple ROIs in the left hemisphere, only those intersecting with the anatomical reference regions described in the previous paragraph were included in the analysis. For Template A, only the anterior language ROI was available based on the previously published study (Gohel et al., 2019); thus, Template A was not included for evaluation in the posterior PLAs.

The following two indices were calculated to compare the templates with fMRI results: (1) the fraction of PLA activations that was within the template, or positive inclusion fraction (PIF):

(1) $$\mathrm{PIF}\;=\;{\left[P_{\mathrm{in}}/\left(P_{\mathrm{in}}\;+\;P_{\mathrm{out}}\right)\right]}_{i}$$

where *P*_in_ is the extent of PLA activation within the template, *P*_out_ is the extent of PLA activation outside of the template, *i* is anterior or posterior, and (2) the fraction of activations within the template that did not belong to PLAs, or false inclusion fraction (FIF):

(2) $$\mathrm{FIF}\;=\;{\left[NP_{\mathrm{in}}/\left(P_{\mathrm{in}}\;+\;NP_{\mathrm{in}}\right)\right]}_{i}$$

where NP_in_ is the extent of non‐PLA activation within the template. Equation (2) was calculated only when there were activations within the template. In addition, Dice similarity coefficient (DSC) (Tie et al., 2014) was used to evaluate the spatial similarity of each language template with the PLA activations of the patients.

### 2.4 Statistical analysis

The nonparametric repeated‐measure Friedman test and post hoc Wilcoxon ranked‐sum test with Bonferroni correction were performed to determine significant differences between templates in the PIFs, FIFs, and DSCs using IBM SPSS version 14.

## 3 RESULTS

Of the 39 patients included, 37 had both LETT and SENT paradigms, and two had one of the paradigms, yielding a total of 76 fMRI datasets that were processed. One dataset was then excluded due to head motions, and three were excluded because no significant activation was detected (using the FWE‐corrected *p *< .05 threshold) in both anterior and posterior PLAs. Among the remaining 72 fMRI datasets (34 LETT and 38 SENT), 62 and 61 had significant activations in anterior and posterior PLAs, respectively. Each of the templates had intersections with the significant activations (PLA or non‐PLA) detected by most of the fMRI datasets (anterior templates: 84%–98%, posterior templates: 82%–97%). All 72 fMRI datasets were also analyzed with the AMPLE threshold.

### 3.1 Comparison of fractional primary language activation across language templates

**FIGURE 2:**
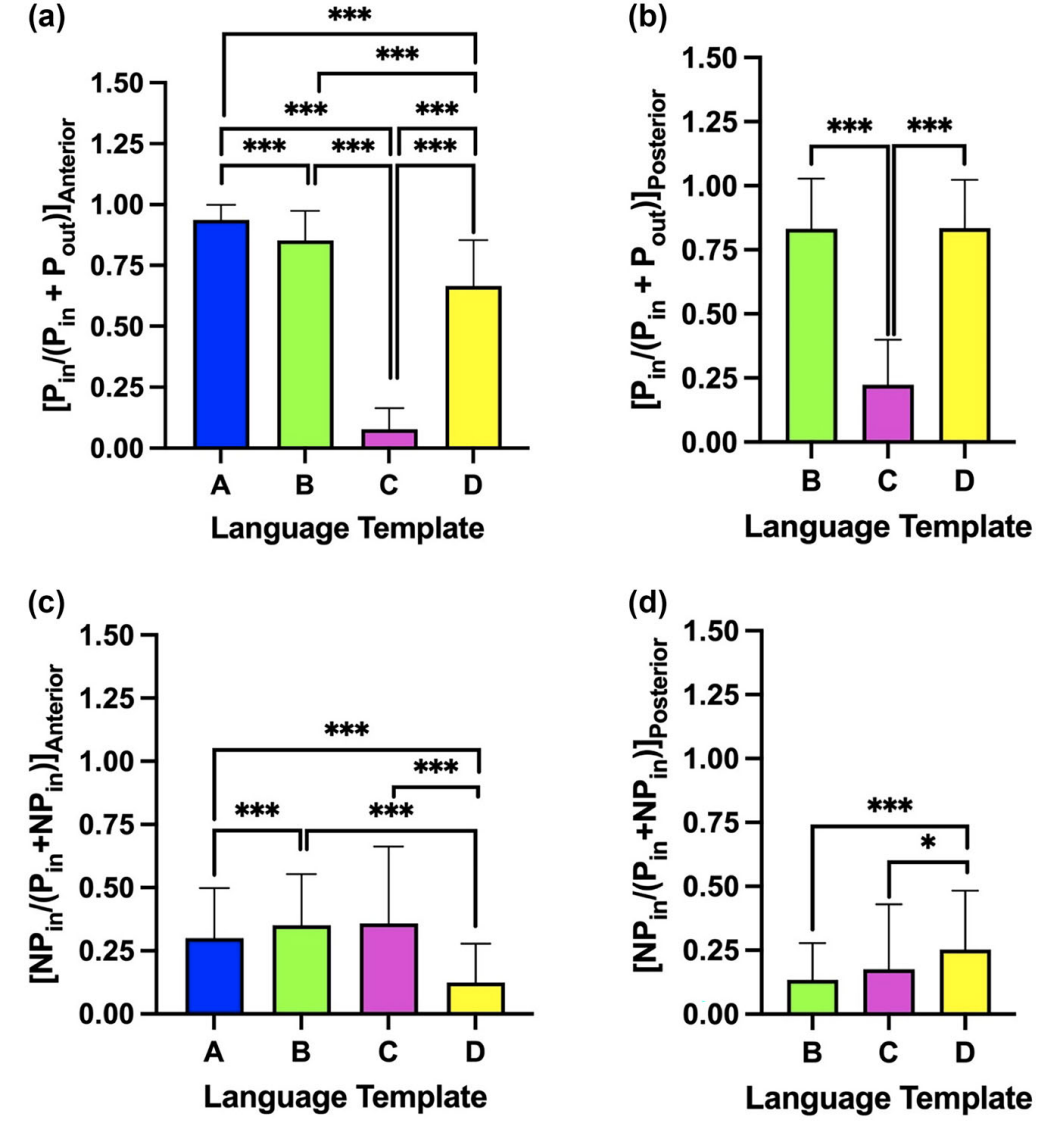
Comparisons of the fractional primary language area (PLA) functional MRI (fMRI) activations (*p* < .01, family‐wise error [FWE] corrected) within the anterior (a) and posterior (b) templates, and of fractional fMRI activations within the anterior (c) and posterior (d) templates that are non‐PLA. The error bars indicate standard errors. *p < .05, ***p < .001. *P*_in_ = Extent of PLA activations within the template. *P*_out_ = Extent of PLA activations outside of the template. NP_in_ = Extent of non‐PLA activations within the template.

Figure 2a,b demonstrates the PIFs of anterior and posterior PLA templates, respectively. Of all the anterior language templates, the mean PIFs for Templates A, B, C, and D were .94 ± .063, .85 ± .12, .077 ± .087, and .67 ± .19, respectively. The Friedman test showed that the PIFs significantly differed from each other (*χ* (Biswal et al., 1995) (3) = 173.44, *p* < .001). Template A enclosed the most amount of anterior PLA activation compared to the other three templates (median, .95; *p* < .001). Across the posterior primary language templates, the mean PIFs were .81 ± .23, .22 ± .19, and .82 ± .22 for Templates B, C, and D, respectively. The Friedman test showed that the PIFs were significantly different from each other (*χ* (Biswal et al., 1995) (2) = 84.45, *p* < .001). Templates B and D enclosed significantly higher fractions of posterior PLA activation compared to Template C (median, .20; *p* < .001), but they did not differ significantly from each other (median, .91 vs. 0.89; *p* = .70).

Figure 2c,d illustrates the FIFs of the anterior and posterior templates, respectively. For the anterior templates, the mean FIFs were .29 ± .15, .35 ± .16, .38 ± .30, and .12 ± .13 for Templates A, B, C, and D, respectively. The Friedman test found that the FIFs were significantly different from each other (*χ* (Biswal et al., 1995) (3) = 72.02, *p* < .001). Template D enclosed the lowest amount of non‐PLA activation compared to the other three templates (median, .074; *p* < .001). For the posterior templates, the mean FIFs were .14 ± .15, .18 ± .26, and .25 ± .22 for Templates B, C, and D, respectively. The Friedman test found that the FIFs were significantly different from each other (*χ* (Biswal et al., 1995) (2) = 15.14, *p* < .001). Templates B and C had similar amounts of non‐PLA activation (median, .061 vs. .054; *p* = .996) but both were lower compared to Template D (median, .19; *p* < .05).

Similar trends were observed with the AMPLE threshold. Template A (.92 ± .14) had the highest PIFs of the anterior PLA. Templates B and D (.80 ± .22 and .80 ± .23, respectively) had significantly higher PIFs of the posterior PLA. Additionally, Template D (.13 ± .15) had the lowest FIFs of the anterior PLA, whereas Templates B and C (.16 ± .20 and .15 ± .26) had the lowest FIFs of the posterior PLA.

### 3.2 Spatial similarity of language template with primary language activation

**FIGURE 3:**
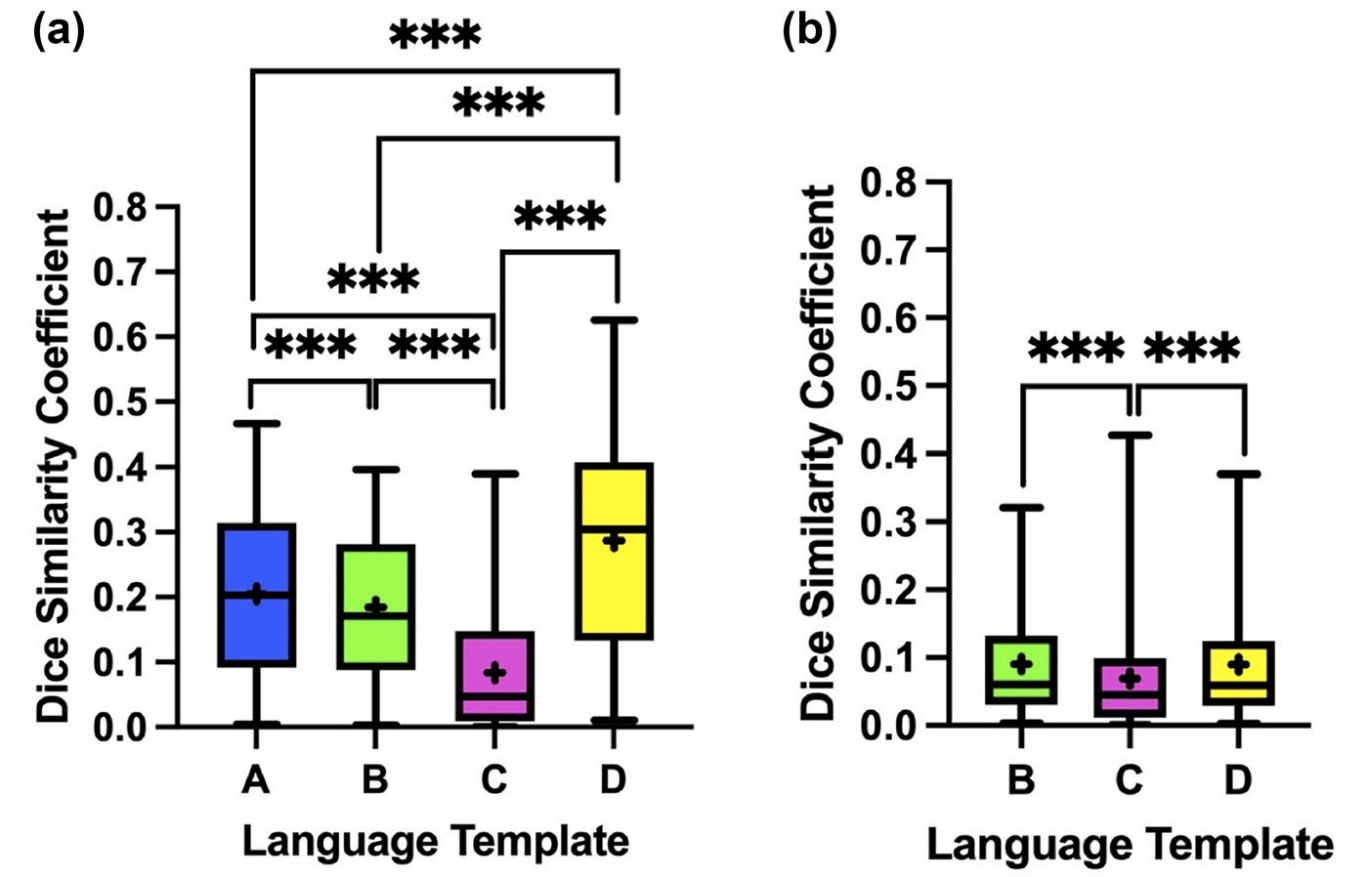
Box‐and‐whisker plot of the Dice similarity coefficients between anterior (a) and the posterior (b) templates and the functional MRI (fMRI) activations (*p *< .05, family‐wise error [FWE] corrected) in patients’ primary language areas (PLAs). The middle line of the box is the median, and the edges indicate the 25th and 75th percentiles. The whiskers are the maximum and minimum of data points and the plus (+) symbol is the mean. ***p < .001.

Figure 3 shows the DSC results of each template and the PLA activations (*p < *.05, FWE corrected). For the anterior primary language templates, the mean DSCs for Templates A, B, C, and D were .20 ± .12, .18 ± .11, .083 ± .091, and .29 ± .17, respectively (Figure 3a). The Friedman test determined that the DSCs were significantly different from each other (*χ* (Biswal et al., 1995) (3) = 131.36, *p* < .001). The post hoc test with Bonferroni correction showed that the DSC of Template D had the highest spatial similarity (median, .30; *p* < .001), whereas Template C had the lowest spatial similarity (median, .047; *p* < .001) with the anterior PLA activation of the patient cohort.

For the posterior primary language templates, the mean DSCs for Templates B, C, and D were .090 ± .081, .069 ± .081, and .090 ± .084 (Figure 3b). The Friedman test determined that the DSCs were significantly different from each other (*χ* (Biswal et al., 1995) (2) = 33.67, *p* < .001). The post hoc test with Bonferroni correction revealed that the DSCs of Templates B and D were significantly higher than that of Template C (median, .045; *p* < .001), but there was no significant difference between Templates B and D (both medians, .059; *p* = .95).

When the AMPLE threshold was used to determine areas with fMRI language activations, similar trends were observed as those described before, when the FWE‐corrected *p *< .05 threshold was used. For the anterior primary language templates, Template D (.27 ± .14) demonstrated the highest spatial similarity to the PLAs, whereas Template C (.088 ± .083) showed the lowest spatial similarity. The differences were statistically significant (*p *< .001). For the posterior primary language templates, Templates B and D (.088 ± .068 and .087 ± .069, respectively) had significantly higher DSCs compared to Template C (.064 ± .067).

**FIGURE 4:**
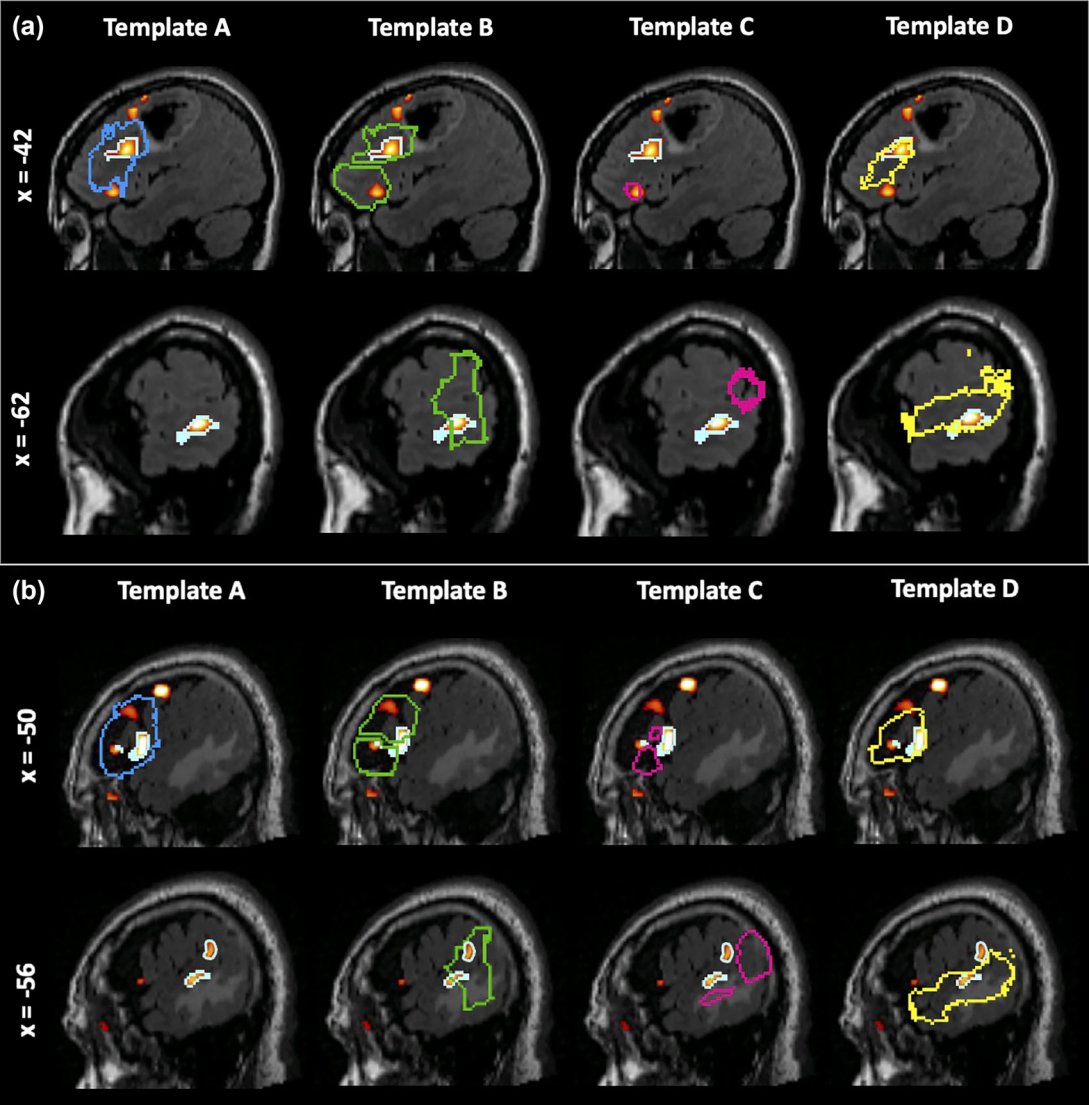
Areas with significant activations (*p *< .05, family‐wise error [FWE] corrected) detected by language functional MRI (fMRI) in two representative patients, (a) and (b), displayed as clusters with colors ranging from red to yellow and white contours, along with four templates overlaid on their normalized T2‐weighted FLAIR images. For each patient, the sagittal slices having the largest extent of anterior (top row) and posterior (bottom row) primary language areas (PLAs) are shown. Blue contour: Template A, green contour: Template B, pink contour: Template C, yellow contour: Template D.

Figure 4 demonstrates each of the four anterior and three posterior language templates overlaid on the normalized T2 FLAIR images of two representative patients. Patient A had an anaplastic astrocytoma located in the left frontal region (Figure 4a), whereas Patient B had a glioblastoma located in the left temporal area (Figure 4b). Template A covered 92% and 97% of the anterior PLAs for Patients A and B, respectively, which were highest among all templates. Template D included the lowest amount of non‐PLA activations for Patients A and B (.77% and 2.2%, respectively). For both patients, the anterior language ROI outlined with Template D most closely resembled that of the classically defined anterior PLA (DSC = .37 and .35, respectively). For the posterior PLAs, Templates B (82% and 85%) and D (94% and 60%) both covered more posterior PLAs for Patients A and B than did Template C (27% and 0%). Templates B and D enclosed the least amount of non‐PLA activation for Patients A and B (0% and 17%), respectively. Templates B (DSC = .12 and .063) and D (DSC = .14 and .043) had higher spatial similarity compared to Template C (DSC = .10 and 0, respectively) for both patients.

## 4 DISCUSSION

Four brain templates were tested to determine the ones that could best represent fMRI primary language activations in brain tumor patients. Overall, among the anterior templates, Template A enclosed most of the anterior PLA activation, whereas Template D had the lowest non‐PLA activation and the highest DSC. Across the posterior primary language templates, Template D enclosed the highest amount of posterior PLA activation, whereas Template B had both the lowest amount of non‐PLA activation and the highest DSC.

It is worth noting that the four templates were generated using distinct approaches and with different cohorts of human subjects. Template A was anatomically determined with the Harvard–Oxford Probabilistic Atlas composed of MR brain images of 37 healthy subjects (Caviness et al., 1996). Template B was generated based on tb‐fMRI studies of 37 healthy subjects after they read sentences versus after they read nonword strings (Fedorenko et al., 2010). Specifically, 13 functional ROIs in the frontal and posterior regions from both the right and left hemispheres representing major language areas sensitive to word‐ and sentence‐level meaning were generated (Fedorenko et al., 2010). Template C was built based on rs‐fMRI studies of 15 healthy subjects. The rs‐fMRI scans were processed using ICA, resulting in 90 functional ROIs representing 14 large‐scale resting state networks (Shirer et al., 2012). Template D was obtained from meta‐analysis results from Neurosynth, using the term “language,” resulting in data from 1101 studies and constrained with anatomical regions defined by the LONI Probabilistic Brain Atlas (Hsu et al., 2020; Yarkoni et al., 2011). When each template was inspected individually, the resulting language ROIs roughly resembled canonical language areas. However, as illustrated by Figure 1, the four templates had different sizes with varied boundaries.

For the purpose of this study, we attempted to resemble each template as they were applied in published clinical studies and focused on the PLAs (Branco et al., 2016; Gohel et al., 2019; Hsu et al., 2020; Zacà et al., 2018). Despite the anterior and posterior language templates were anatomically constrained to the PLAs, they differed from each other due to their various sizes and how much of the PLAs were covered. Because of the variability of the templates, their use for clinical fMRI analysis will likely depend on the needs of specific applications. For example, if the application requires covering as much of the potential primary language activation as possible, that is, a higher PIF, templates with larger spatial extents would be ideal. For this purpose, Template A would be ideal for the anterior language area and Template D for the posterior language region. This recommendation would concur with a recent report of expansion of the typical definitions of primary anterior and posterior language areas to include areas slightly beyond the Sylvian fissure (Sanai et al., 2008). However, the disadvantage to having a template that is bigger than the traditionally defined PLAs is that there is a higher chance of including fMRI activations that are not specific to PLAs, that is, a lower FIF. Therefore, if the goal is to cover only PLA activations specifically, Template D would be ideal for the anterior PLA, and Template B would be ideal for the posterior PLA. It is not intuitive which template characteristics are preferred for different applications. For example, if one speculates that language lateralization is similar between PLA and non‐PLA activations, a larger template could be desirable for the laterality calculation. On the other hand, for rs‐fMRI analyses, if PLAs and non‐PLAs may not be functionally connected, one may prefer using templates that are more specific to PLAs to detect the primary language network. Therefore, more application‐specific studies will be needed to determine the template selection.

It is interesting to note that Template C, which was generated based on rs‐fMRI, did not outperform other templates in any of the comparisons. We speculate that the result was due to the smaller extents of the template and/or the small sample from which the template was derived, which could not account for the spatial variation of PLAs in our patient population.

As our sample consisted of patients with tumors located on both the right and left hemisphere, we considered potential tumor laterality effects where brain tumors can cause a shift of language laterality to the nondominant hemisphere (Giussani et al., 2010; Krieg et al., 2013; Thiel et al., 2005). First, this study only focused on language activation on the left hemisphere. Second, the average result of 4/39 patients with tumors on the right hemisphere adhered to the overall trend of our results and did not affect the conclusion of our paper. Furthermore, we also considered the language dominance that can be related to the handedness of patients (Knecht, 2000). Among the 39 patients included in this study, 32 had right‐handed dominance, 5 had mixed, and 2 had left‐handed dominance. A total of 35/39 of our patients had language dominance in the left hemisphere, 3 had codominance, and 1 had right‐hemisphere language dominance. The average result of the four patients with non‐left hemisphere language dominance followed the overall trend of the conclusion of this paper. However, due to the small sample sizes of the patients with right‐hemisphere tumors, left‐handed dominance, or right‐hemisphere language dominance in this study, we are not able to investigate these effects further.

The DSCs between the templates and fMRI activations were generally low. This is not surprising because templates are not based on measurements in individuals, in addition to other factors as discussed below. First, our subjects were brain tumor patients with lesions located mainly in or adjacent to the anterior and/or posterior language areas, which might have resulted in compromised language activations due to anatomical/functional distortion or neurovascular uncoupling (Agarwal et al., 2016). Additionally, it is not uncommon that the templates would even partly overlap with lesions when applied to patients with brain tumors. Second, standard thresholding approaches (i.e., FWE correction and AMPLE) were applied across all patients despite variability in their fMRI performance. Third, the pattern of fMRI activations depends on the nature of task paradigms and the patient's performance of the paradigms. An additional contributing factor to the low posterior language DSC, specifically, is that the posterior templates have much larger spatial extents than the actual fMRI activations. The distribution of the posterior PLA has been reported to be more varied (Tie et al., 2014) compared to its anterior counterpart that could partly explain for the posterior language templates needing to be larger. Considering these factors, although DSC is an interesting metric to help understand the spatial similarity between templates and fMRI activation, the other metrics such as PIF and FIF may be more useful to assess the usefulness of the templates.

The LETT and SENT paradigms used in this study are the top two paradigms recommended by the ASFNR for presurgical language fMRI (Black et al., 2017). SENT is a robust clinical paradigm that activates both anterior and posterior PLAs, whereas LETT involves more anterior than posterior PLAs (Barnett et al., 2014; Zacà et al., 2013). It is quite common to use the two paradigms combinedly for clinical interpretation such as in our institution. Although both paradigms are expected to involve the same PLAs, the activation extent varies based on the nature of the language tasks as well as the patient performance of the tasks. This is an important favor to consider when interpreting the results of this study as well as applying templates in clinical fMRI analysis.

In addition to the PLA analyzed in this study, other language areas are important for supporting essential speech and may need to be avoided during tumor resection (Brennan et al., 2016). For example, direct cortical stimulation of the superior and posterior middle frontal gyrus in patients undergoing tumor resection yielded positive findings, including speech arrest and anomia (Hazem et al., 2021). Additionally, damage to the supplementary motor area has been shown to cause motor and/or speech deficits (Brennan et al., 2016; Hazem et al., 2021). Further studies on brain templates of these language areas will be needed for applications focusing on mapping these areas for presurgical evaluation.

### 4.1 Limitations

This study has a limited scope of comparing four templates with two clinical fMRI paradigms in the left traditionally defined PLAs. Thus, the results may not be generalized to other templates, with different paradigms, and in other language‐associated brain areas. The four templates were selected because they had been used in published clinical fMRI studies and they are publicly available (Branco et al., 2016; Gohel et al., 2019; Hsu et al., 2020; Zacà et al., 2018). These templates were not built based on brain tumor populations, although they have been applied in language mapping studies in brain tumor patients. The two fMRI paradigms were limited to those used in our institution, but they were consistent with the ASFNR recommendations for presurgical language assessment (Black et al., 2017). Only the left hemisphere was evaluated, which means that we cannot generalize our findings to people with language dominance on the right side of the brain. Studying other language areas than the left PLAs in brain tumor patients is underway and will need to involve different functional templates dedicated to these brain areas (Giussani et al., 2010; Krieg et al., 2013; Thiel et al., 2005).

Historically, the classical model of language network consisted of Broca's and Wernicke's areas that were based on clinical observations of patients with aphasia (Binder et al., 1997; Gohel et al., 2019; Hertrich et al., 2020; Tie et al., 2014). Despite they have been commonly referred to as PLAs in the clinic (Binder et al., 1997; Hertrich et al., 2020), there is no universal agreement on the specific cortical areas that make up these core language networks (Binder et al., 1997). For example, Wernicke's area can be defined as including the angular gyrus, supramarginal gyrus, and the posterior half of superior temporal gyrus of the left temporal cortex (Talairach & Tournoux, 1988), whereas others consider Wernicke's to be within the superior and middle temporal regions (Hertrich et al., 2020). Therefore, due to the variability in the cortical components that make up the PLAs, a definition of the PLA that differs from ours could yield different results. This is another limitation of the present study.

Furthermore, this was a single‐institution study with the fMRI paradigms performed by brain tumor patients. Although our results are likely applicable for most of the brain tumor population undergoing presurgical evaluation of PLAs, more studies are needed to increase the generality of the utilities of language brain templates.

## 5 CONCLUSION

Four publicly available language brain templates that have been applied in clinical fMRI studies were found to vary in spatial extent among themselves and to exhibit different inclusiveness of and spatial agreements with fMRI PLA activations in brain tumor patients. Templates A and D captured the most amount of primary language activation in the anterior and posterior language area, respectively. Templates D and B included a least amount of non‐PLA activations in the respective areas. They also had the highest spatial similarity with fMRI activations in the anterior and posterior PLAs, respectively. These features can be used to guide the selection of language templates for clinical fMRI analysis Figure S1.

## AUTHOR CONTRIBUTIONS

**Jina Lee**: Conceptualization; methodology; software; data curation; investigation; validation; formal analysis; visualization; project administration; writing—original draft; writing—review and editing. **Vinodh A. Kumar**: Conceptualization; investigation; methodology; validation; formal analysis; visualization; writing—original draft; writing—review and editing. **Jian Ming Teo**: Methodology; Software; data curation; investigation; validation; formal analysis; visualization; writing—review and editing. **Rami W. Eldaya**: Validation; formal analysis; visualization; writing—review and editing. **Ping Hou**: Data curation; writing—review and editing; methodology. **Kyle R. Noll**: Conceptualization; data curation; writing—review and editing. **Sherise D. Ferguson**: Conceptualization; data curation; writing—review and editing. **Sujit S. Prabhu**: Conceptualization; data curation; writing—review and editing. **Ho‐Ling Liu**: Conceptualization; methodology; investigation; data curation; validation; formal analysis; supervision; funding acquisition; visualization; project administration; resources; writing—original draft; writing—review and editing.

## CONFLICT OF INTEREST STATEMENT

The authors declare no conflicts of interest.

## FUNDING INFORMATION

NIH/NCI under, Award Numbers: R01 CA258788 and P30 CA016672

### PEER REVIEW

The peer review history for this paper is available at https://publons.com/publon/10.1002/brb3.3497

## Supporting information




**Figure S1**. T1‐weighted images of a representative patient before spatial normalization (a) and after spatial normalization (b), in comparison to the MNI standard T1‐weighted image (c).

## Data Availability

The data that support the findings of this study are available on request from the corresponding author.
