# Supplementary figures and images for: Comparative analysis of brain language templates with primary language areas detected from presurgical fMRI of brain tumor patients
Source: Brain Behav. 2024 Jun 19;14(6):e3497. doi: 10.1002/brb3.3497 (PMC11186848; doi:10.1002/brb3.3497)

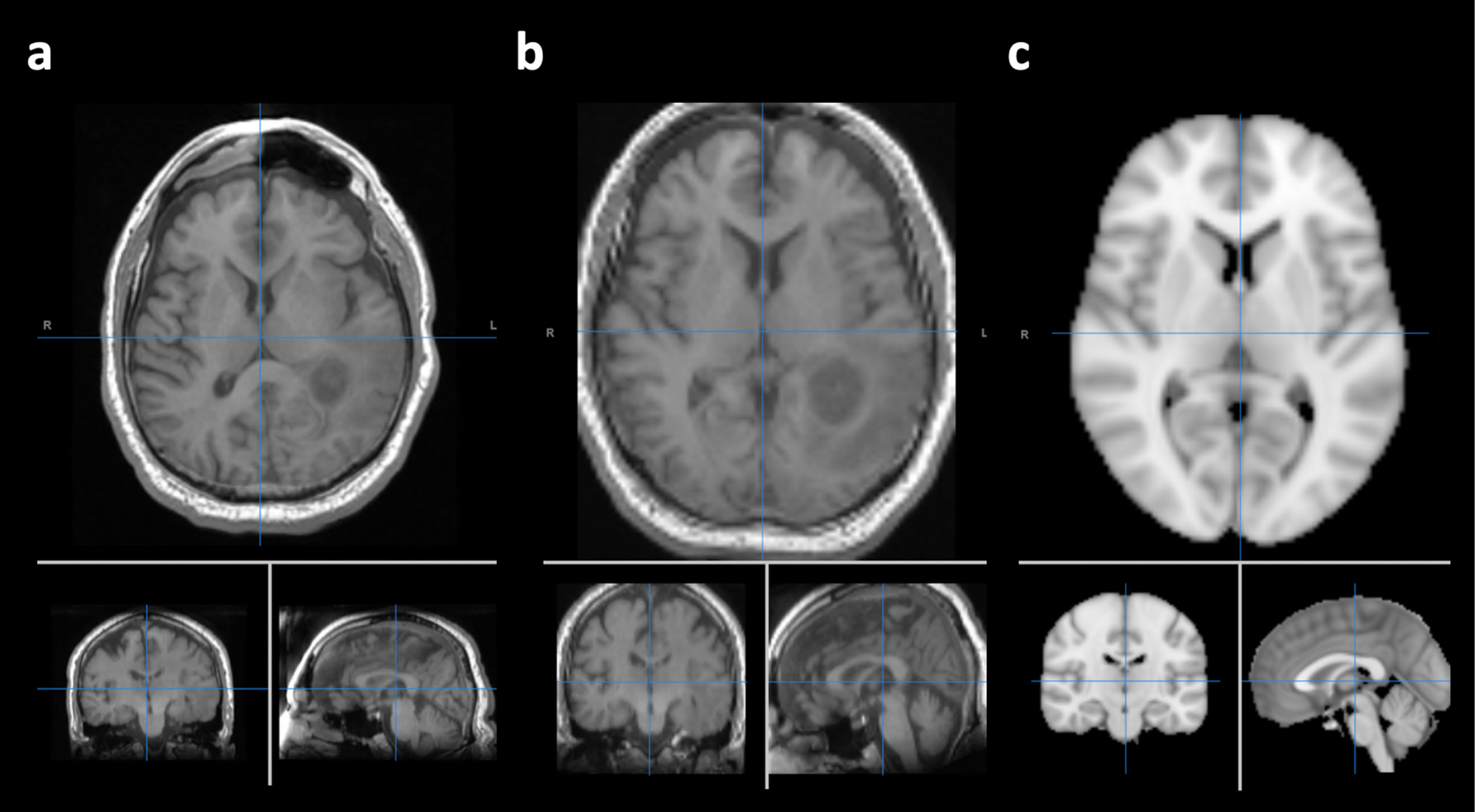

Supplement: Supplementary file 1 — Figure S1. T1‐weighted images of a representative patient before spatial normalization (a) and after spatial normalization (b), in comparison to the MNI standard T1‐weighted image (c). [file BRB3-14-e3497-s001.png]
